# Understanding microglial responses in large animal models of traumatic brain injury: an underutilized resource for preclinical and translational research

**DOI:** 10.1186/s12974-023-02730-z

**Published:** 2023-03-09

**Authors:** Michael R. Grovola, Catherine von Reyn, David J. Loane, D. Kacy Cullen

**Affiliations:** 1 Center for Neurotrauma, Neurodegeneration & Restoration, Corporal Michael J. Crescenz VA Medical Center, Philadelphia, PA USA; 2 Department of Neurosurgery, Center for Brain Injury & Repair, University of Pennsylvania, 105E Hayden Hall/3320 Smith Walk, Philadelphia, PA 19104 USA; 3 School of Biomedical Engineering, Science and Health Systems, Drexel University, Philadelphia, PA USA; 4 Department of Neurobiology and Anatomy, Drexel University College of Medicine, Philadelphia, PA USA; 5 School of Biochemistry and Immunology, Trinity College Dublin, Dublin, Ireland; 6 Department of Anesthesiology and Shock, Trauma, and Anesthesiology Research (STAR) Center, University of Maryland School of Medicine, Baltimore, MD USA; 7 Department of Bioengineering, School of Engineering and Applied Science, University of Pennsylvania, Philadelphia, PA USA

**Keywords:** Mild TBI, Neuroinflammation, Microglia, Large animal models, Preclinical models

## Abstract

Traumatic brain injury (TBI) often results in prolonged or permanent brain dysfunction with over 2.8 million affected annually in the U.S., including over 56,000 deaths, with over 5 million total survivors exhibiting chronic deficits. Mild TBI (also known as concussion) accounts for over 75% of all TBIs every year. Mild TBI is a heterogeneous disorder, and long-term outcomes are dependent on the type and severity of the initial physical event and compounded by secondary pathophysiological consequences, such as reactive astrocytosis, edema, hypoxia, excitotoxicity, and neuroinflammation. Neuroinflammation has gained increasing attention for its role in secondary injury as inflammatory pathways can have both detrimental and beneficial roles. For example, microglia—resident immune cells of the central nervous system (CNS)—influence cell death pathways and may contribute to progressive neurodegeneration but also aid in debris clearance and neuroplasticity. In this review, we will discuss the acute and chronic role of microglia after mild TBI, including critical protective responses, deleterious effects, and how these processes vary over time. These descriptions are contextualized based on interspecies variation, sex differences, and prospects for therapy. We also highlight recent work from our lab that was the first to describe microglial responses out to chronic timepoints after diffuse mild TBI in a clinically relevant large animal model. The scaled head rotational acceleration of our large animal model, paired with the gyrencephalic architecture and appropriate white:gray matter ratio, allows us to produce pathology with the same anatomical patterns and distribution of human TBI, and serves as an exemplary model to examine complex neuroimmune response post-TBI. An improved understanding of microglial influences in TBI could aid in the development of targeted therapeutics to accentuate positive effects while attenuating detrimental post-injury responses over time.

## Background

Traumatic brain injury (TBI) is a serious health concern and a major contributor to morbidity and mortality in the United States each year. The most recent surveillance report from the Centers for Disease Control and Prevention described estimates of the national incidence of TBI from 2006 to 2014: the CDC reported 2.87 million patients diagnosed with TBI and 56,800 TBI-related deaths in 2014 alone [1]. TBI is typically caused by a mechanical insult, such as a fall or motor vehicle crash, which impacts and/or rapidly accelerates the head (typically angular rotation) producing focal contusions, hematomas, and/or shear deformation of white matter tracts [2]. This primary physical insult may induce a number of secondary injuries, such as blood–brain barrier (BBB) permeability, neuronal dysfunction, dendritic/synaptic disruption, astrocyte reactivity, leukocyte infiltration, axonal degeneration, cell death, and neuroinflammation; all of which are responsible for neurological impairments following TBI [2–6].

In particular, neuroinflammation has gained increasing attention for its role in secondary injury as neuroinflammatory responses can have both detrimental and beneficial roles. Microglia—the resident immune cell of the central nervous system (CNS)—mediate cell death and may contribute to progressive neurodegeneration but also aid in debris clearance and neuroplasticity [7]. Further complicating the role of microglia, their activity is influenced by patient age, sex, mechanism of injury, degree of injury, co-morbidities, and genetic factors [8]. Insight into these inflammatory profiles and their evolution over time is crucial to the development of treatment strategies to manage the neuroinflammatory deficits produced by TBI. Here, we will review the current understanding of the roles of microglia following TBI based on experimental and clinical reports, with a particular focus on recently described chronic neuroinflammatory responses in an established porcine model of closed-head diffuse TBI.

## Development and role of microglia

Microglia are parenchymal immune cells that are distinct from perivascular, meningeal, and choroid plexus macrophages in the CNS due to their cell lineage. Microglia are formed during primitive hematopoiesis, where macrophages derived from the yolk sac migrate down the neural tube and eventually become microglia [9]. In contrast, other CNS macrophages are derived from hematopoietic stem cells in the bone marrow and require the Myb transcription factor for proper development [10]. As such, microglia are genetically distinct from other CNS macrophages, which would in turn imply functional differences between the two cell populations. Indeed, microglia have been shown to play crucial and distinct functional roles in the brain. Numerous studies have demonstrated that microglia have noninflammatory, homeostatic functions in the healthy brain. Microglia have sentinel-like activity; they continually scan their local environment and utilize their processes to contact and monitor neurons [11, 12]. This continuous surveillance covers the entire brain over the course of several hours, possibly enabling microglia to clear the parenchyma of accumulated metabolic products and deteriorated tissue components [12].

Though the precise role of microglia process motility and surveillance is unclear, one of microglia’s most likely tasks is to influence synaptic function in both healthy and diseased states through the process of synaptic pruning. During early neuronal development, microglia engulf axons and synaptic components to shape neuronal circuits [13, 14]. Super resolution microscopy in the mouse hippocampus has shown colocalization of microglia with both PSD95, an excitatory postsynaptic marker, and SNAP25, a presynaptic protein, during the period of synaptic maturation. In addition, delaying this synaptic pruning resulted in an excess of dendritic spines and electrophysiologically immature brain circuitry [14]. Interestingly, in the mouse visual system, synaptic pruning by microglia is regulated by neural activity, as significantly more synaptic inputs were engulfed after treatment with Tetrodotoxin, an action potential inhibitor, while significantly less synaptic inputs were engulfed after treatment with forskolin, which increases cAMP and neural activity. The microglia-specific phagocytic complement pathway may underlie some synaptic changes, yet knocking out complement receptors (CR3) only reduced pruning by 50% suggesting that other phagocytic mechanisms are also involved [13]. Challenging the extent of synaptic remodeling, Weinhard et al. directly tested this role of microglia in phagocytic synapse elimination in organotypic mouse hippocampal cultures. Employing time-lapse light sheet microscopy on microglia-synapse interactions, they found partial elimination of presynaptic boutons and axons by microglia, but no elimination of postsynaptic material. Instead, microglia reorganized postsynaptic sites by inducing spine filopodia formation [15]. In addition, microglia may alter activity-dependent visual circuitry; for instance, the prolonged closure of one eye changes connections reserved for binocular vision yet disrupting the P2Y12 purinergic receptor expressed in microglia while closing one eye negates these changes [16]. Future research will be needed to examine this more nuanced role for microglia in synapse remodeling as well as assess how specific synapses are recognized and targeted for synaptic pruning.

While changes to microglia can impact neural circuits during development, learning and memory can also be impaired due to microglial changes in adulthood. Indeed, in the healthy adult CNS microglia appear to be involved in activity-dependent long-term synaptic plasticity. One recent study found that experience-driven accumulation of neuronal interleukin(IL)-33 directs microglia to engulf the extracellular matrix, and loss of IL-33 leads to reduced plasticity and diminished fear memory integration [17]. Moreover, knocking out the fractalkine receptor, a necessary receptor for neuron–microglia communication, has led to deficits in contextual fear conditioning, spatial learning and memory, and long-term potentiation (LTP) [18].

Microglia’s impact on learning and memory is finely interwoven with microglia’s regulation of neurogenesis. In the adult hippocampus, a structure essential for memory formation, neuroprogenitor cells produce neuroblasts in the dentate gyrus. However, the majority of these cells do not integrate into hippocampal circuitry as many cells undergo apoptosis and are phagocytosed by microglia [19]. In fact, during both early development and ongoing adult neurogenesis, microglia phagocytose neurons that die as a result of programmed cell death (for a review see [20]). These phagocytic functions are regulated by TAM receptor tyrosine kinases Mer and Axl, as mice deficient in these kinases exhibit an accumulation of apoptotic cells [21]. Yet microglia also play an active role in apoptosis by releasing superoxide ions or tumor necrosis factor (TNF), indicating that microglia can also drive programmed cell death and further diversifying their physiological roles in the brain [22–24].

## Phenotypes of microglia

Microglia are dynamic cells with a wide repertoire of functions that respond to changes in their microenvironment. Traditional microglial evaluation attempted to classify them into a pro-inflammatory (M1) or anti-inflammatory (M2) bimodal arrangement, with each having their own phenotypic markers. M2 microglia were thought to be responsible for neurogenesis regulation and synaptic plasticity, and express IL-10, TGFβ, CD206, Arg-1, whereas M1 were thought to be stimulated by DAMPs, responsible for long-term inflammation and neurodegeneration, and express IL-1β, TNF, IL-6 [8]. However, transcriptomic profiling does not support this hypothesis and has led to a rejection of a M1 vs M2 dichotomy. For instance, acutely isolated microglial cultures from injury models have cells which co-express both sets of these “signature” genes or express none of these genes [25, 26]. Based on these studies, the M1 vs M2 classification appears to be overly simplistic and insufficient to classify heterogenous sets of microglia. Indeed, a 2022 white paper written by leading microglial researchers discard this M1 vs M2 labeling and provide a series of recommendations for diverse microglial nomenclature [27].

Accordingly, in an effort to distinguish microglia subpopulations in different states, one study examined more than 76,000 individual mouse microglia during development, old age, and after brain injury. Their analysis detected at least nine transcriptionally distinct microglia states defined by unique markers [28]. In 2022, Shih et al. conducted the first study to define the pig microglial transcriptome and conduct interspecies comparisons. They found 239 core microglial genes as well as 150 genes that varied based on brain region. In addition, normalized gene expression was compared to humans and rodents, and demonstrated that core microglial genes are conserved across species, while species-specific expression also exists [29]. By highlighting the complexity of transcriptional microglia signatures, the field is gradually taking steps towards elucidating the function of numerous microglial states. Future studies will need to test each putative state individually, possibly using genetic manipulation to alter each subpopulation to eventually determine their function and phenotype.

## Extracellular injury signals that drive microglia

After TBI, extracellular signals trigger microglia to perform a variety of both neurodegenerative and regenerative functions. TBI may induce membrane permeabilization and/or blood–brain barrier disruption, which can release proteins, nucleic acids, or other molecules collectively known as damage-associated molecular patterns (DAMPs) [8, 30]. One classic example of DAMP release is high mobility group protein B1 (HMGB1); HMGB1 stabilizes nucleosomes under normal conditions but is greatly upregulated after injury and associated with elevated intracranial pressure in TBI patients as well as cerebral edema after moderate TBI in mice [31]. In addition, adenosine 5′-triphosphate (ATP) may be released from damaged neurons into the extracellular environment; ATP triggers a rapid convergence of microglial processes towards the site of injury [11, 32]. Moreover, astrocytes can actively release ATP into the extracellular environment to potentially amplify the immune cell response [11, 33].

**Fig. 1:**
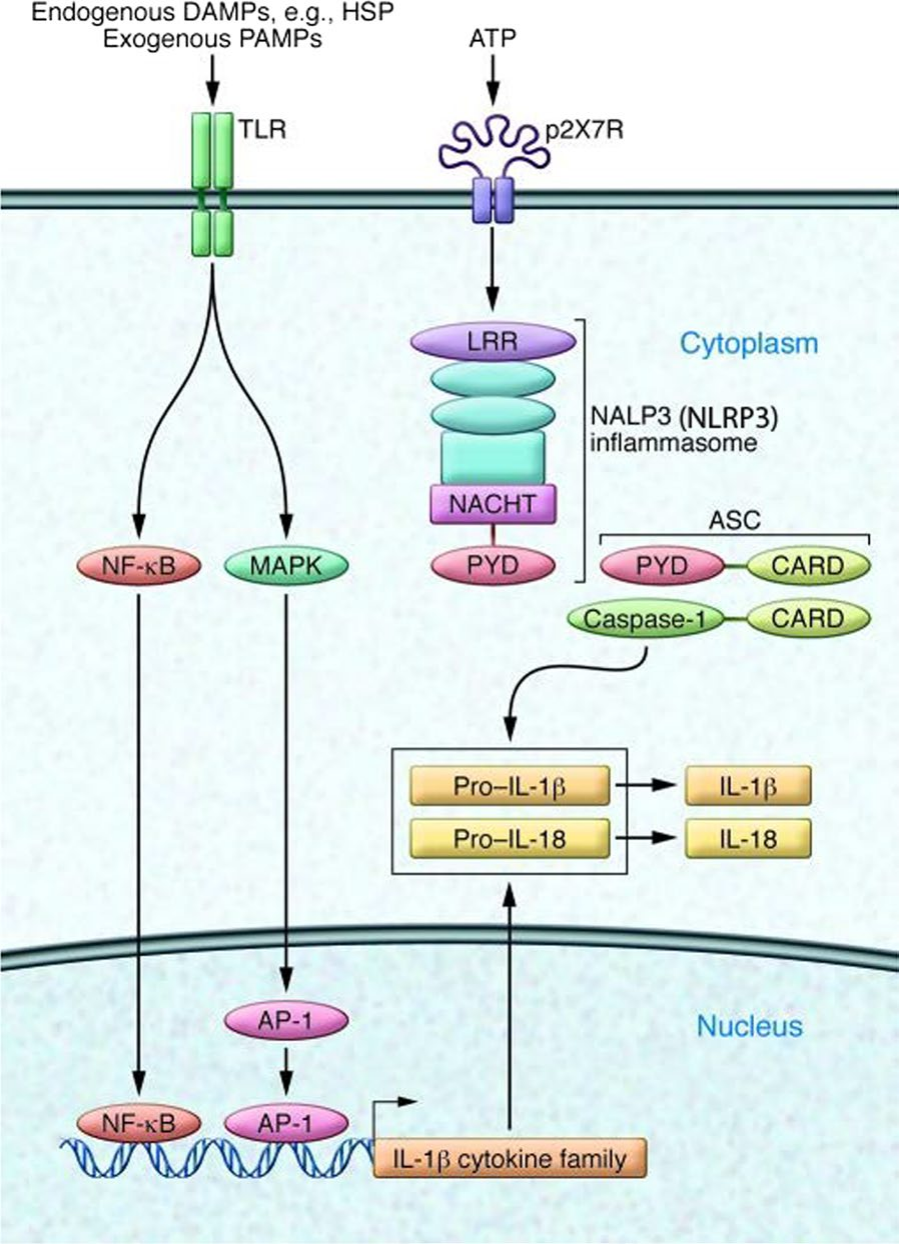
Innate recognition of tissue injury. DAMPs activate cell surface toll-like receptors (TLRs) and ligation of TLRs initiates an intracellular signaling cascade that drive the production of IL-1 family cytokine precursor proteins. ATP activates NOD-like receptors (NLRs), which signals the cleavage and maturation of IL-1 family cytokines (Adapted from Ransohoff and Brown [34])

Detecting post-traumatic DAMP release, cell surface toll-like receptors (TLR) activate microglia and astrocytes, and rapidly upregulate major drivers of neuroinflammation: TNF, IL-6, and IL-1β [34]. Alternatively, ATP can activate NOD-like receptors (NLR), a central part of the inflammasome. Recent studies indicate moderate/severe TBI upregulates the NLRP3 inflammasome, which is increasingly studied as its inhibition improved outcomes in rodent TBI and may serve as a biomarker of inflammatory conditions [35, 36]. Finally, activation of NLRP3 autoactivates caspase-1 and catalyzes the active form of IL-1 family cytokines (Fig. 1) [34].

In parallel with DAMP activity, excessive extracellular glutamate, the major excitatory neurotransmitter of the CNS, may occur. In the healthy brain, astrocytes can uptake excess glutamate released from neurons; however, after TBI, glutamate uptake by astrocytes can be transiently impaired following the injury [37, 38]. This excess glutamate can lead to excitotoxicity via overactivation of *N*-methyl-d-aspartate (NMDA) and α-amino-3-hydroxy-5-methyl-4-isoxazolepropionic acid (AMPA) receptors. Activation of these receptors allows an influx of calcium through receptor-gated channels, voltage-gated channels, or intracellular calcium stores, which can produce proteases, lipases, oxygen radicals, mitochondrial damage, DNA degradation, and ultimately lead to cell death [39].

Microglia themselves express both ionotropic glutamate receptors (iGluRs) and metabotropic glutamate receptors (mGluRs). Following acute CNS injury, local glutamate activity can rapidly alter microglial functional responses. For example, microglia express both AMPA and ainite iGluRs, which upon receptor activation, triggers the release of TNF-α in cultured microglia [40]. Moreover, depending on the different subtypes of mGluRs expressed, microglia may adopt a more neuroprotective or a more neurotoxic phenotype, via the activation of group III and group II mGluRs, respectively [41–43]. The direct activation of group II mGluRs via mGluR2 stimulation induces metabolic stress leading to release of TNF-α and Fas ligand, which triggers neuronal caspase-3 activation via TNFR1 and Fas receptor, leading to neuronal cell death [43]. Furthermore, the link between glutamate toxicity and inflammatory cytokines has been outlined in many studies. For example, excitotoxic brain damage increases TNF-α and IL-1β expression, which in turn increases NMDA receptor-induced neuronal death (for a review see [44]). Further solidifying this connection, NMDA receptor antagonists reduce levels of IL-1β and TNF-α, and IL-1 receptor antagonists reduce excitotoxicity [44]. Unfortunately, NMDA receptor antagonists have failed clinical trials for treatment of TBI patients [45].

## Effects of chronic inflammation

After the acute phase of injury, the optimal outcome is resolution of the inflammatory response and release of trophic factors [7]. However, a subset of patients or animals experience persistent inflammation out to chronic timepoints [46]. One rodent study subjected adult mice to a single moderate controlled cortical impact injury and detected highly reactive microglia up to 1-year post-injury with microglia expressing major histocompatibility complex class II (CR3/43), CD68, and NADPH oxidase (NOX2). Moreover, these biochemical markers were associated with progressive lesion expansion, hippocampal degeneration, and loss of myelin, supporting a link between chronic microglia activation and neurodegeneration [47]. Indeed, there is evidence of persistent inflammation and degeneration after single moderate/severe TBI in humans. Johnson et al. [5], found cases up to 18-years post-injury with CR3/43 and/or CD68 immunoreactive pathology paired with white matter degeneration. Neuroimaging studies (TSPO - Translocator Protein 18kDa) and serum biomarker (TNFα) analysis corroborate this neuropathology of chronically elevated cytokines and inflammation [48, 49].

These analyses have been complimented by RNA sequencing techniques, yielding rich data sets on chronic inflammatory markers, revealing the complexity of microglial heterogeneity after TBI, and enabling more nuanced classification schemes beyond simplistic M1 vs M2 markers. After 1.2 atmosphere midline FPI, Witcher et al. [50] employed single-cell RNA sequencing in mouse cortex at 7 days-post injury (DPI), a critical point in the transition of acute to chronic pathology. They found 10 distinct microglial subclusters determined by each cell’s transcriptome along with increased type-1 interferon and neurodegenerative-related genes. In addition, Witcher et al. utilized nanoString mRNA panels on 30 DPI mouse cortex and found an increase in several transcripts related to innate immunity (CD14, CD68, GPR84, Itgax, TLR4, and TREM2) compared to controls. Todd et al. [51] conducted bulk RNA sequencing on FACS sorted microglia and astrocytes at 7 DPI following severe fluid percussion injury in mice. They identified 518 differentially expressed genes between TBI and sham microglia, with many of the top differentially expressed genes being a part of the type I interferon pathway. Finally, Lipponen et al. [52] examined chronically altered gene expression at 3-months post-injury after 3.3 atmosphere lateral fluid percussion in rats. Using gene ontology analysis, they found immunity and inflammatory genes sets upregulated in the perilesional cortex and thalamus, but not the hippocampus, indicating functional gene sets remain activated over a wide post-TBI time window. Overall, the field is still unsure of the genetic susceptibilities, molecular triggers, and pathways that contribute to chronic inflammation, yet additional factors such as injury severity, sex, and model species are thought to influence inflammation.

## Effects of injury severity and interspecies variation on inflammation

Following injury, microglial activation can persist in the brain for weeks, years, or even decades, as demonstrated in chronic assessments of mild TBI in rodents, chronic assessments of moderate-to-severe TBI in humans, and acute assessments of mild TBI in pigs. However, until recently, the microglial activation dynamics after mild TBI had not been assessed at chronic timepoints in a large animal model. While several large animal models of TBI—most notably porcine models—are in use in the field, such carefully controlled laboratory models may ultimately inform future treatment for humans [53–59].

**Fig. 2:**
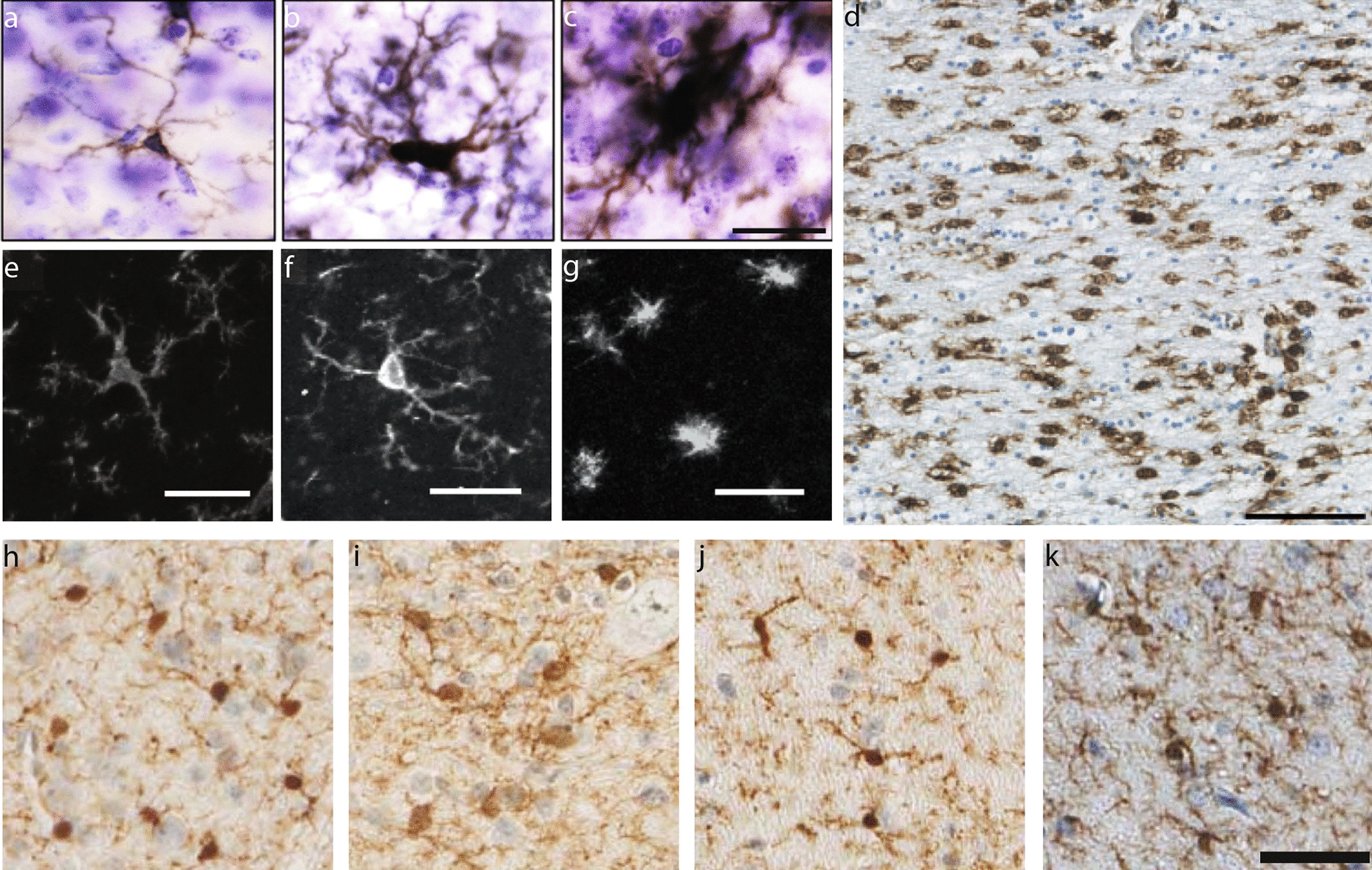
Microglial phenotypes in post-mortem TBI immunochemistry. The pattern and extent of microglial reactivity may vary according to species and injury severity. Representative images of Iba-1+ microglia display ramified (**a**), hypertrophic (**b**), and bushy (**c**) morphologies after moderate-level controlled cortical impact in rodents (scale = 25 µm; images adapted from Henry et al. [62]). Human post-mortem studies demonstrate morphologically altered CR3/43+ microglia after a single moderate-to-severe TBI (**d**) (scale = 100 µm; images adapted from Johnson et al. [5]). In a closed-head rotational acceleration model of TBI in pigs, Iba-1+ microglia display ramified morphologies in sham conditions (**e**) and after acute mild TBI absent permeabilized neurons (**f**) yet display ameboid morphology after acute mild TBI localized with permeabilized neurons (**g**) (scale = 25 µm; images adapted from Wofford et al. [59]). In this same model, Iba-1+ microglia have been shown to undertake a more ramified phenotype after single mild TBI at longer timepoints post-injury. Representative images of Iba-1+ microglia compare morphology in sham (**h**) and 7-day postinjury (**i**) in the hippocampal hilus, and sham (**j**) and 1-year post-injury (**k**) in the hippocampal molecular layer (scale = 50 µm; images adapted from Grovola et al. [67])

Accordingly, a survey of previous work shows that the mechanism(s) and severity of traumatic loading as well as the species being assessed can impact the pattern and extent of neuroinflammation (Fig. 2). Rodent studies have shown a range of chronically altered microglia—with morphologies described as ramified, hypertrophic, or bushy (Fig. 2a–c)—and inflammatory gene expression [2, 47, 60–62]. For example, in a weight drop model of mild TBI, MAPT, GFAP, and TNF genes increased in the cortex at acute time points, while AIF1, CCL11, and TARDBP genes were upregulated in the cortex at chronic timepoints [61]. Comparatively, mice subjected to moderate controlled cortical impact displayed an upregulation of genes in the cortex related to the NLRP3 inflammasome and NADPH oxidase pathways at chronic timepoints [62]. After closed-head rotational acceleration TBI in pigs, microglia display ameboid morphologies around permeabilized neurons, while microglia display more ramified morphologies absent permeabilized neurons and in shams (Fig. 2e–g) [59]. Moreover, microglia reactivity has been shown to vary across species when all subjects are injured using the same model. Gorse and Lafrenaye [63] investigated thalamic microglial–axonal interaction differences between pigs and rats after central fluid percussion injury. They noted a decrease in microglial interactions with injured axons in rats but an increase in microglial interactions with injured axons in pigs suggesting that the species used for in vivo preclinical studies influences post-TBI microglial response.

Human post-mortem studies have described persistent neuroinflammation with morphologically altered microglia after a single moderate-to-severe TBI (Fig. 2d) or after repetitive mild TBIs [5, 64, 65]. These pathological studies often note neuroinflammation in the corpus callosum; however, a neuroimaging study using positron emission tomography (PET) reported inflammation-related ligand binding differences in the thalamus and putamen, but not the corpus callosum after single moderate-to-severe TBI [66]. Interestingly, neuropathological analysis from our lab did not observe microglial reactivity in the corpus callosum after single mild closed-head TBI [67]. Moreover, NFL players with a history of TBI displayed increased TSPO binding (expression of which increases in activated microglia) in the hippocampus, left entorhinal cortex, parahippocampal cortex, supramarginal gyrus, and left temporal pole [48]. Yet, drawing conclusions from these human studies is difficult as these are not able to be as carefully controlled as preclinical studies. Collectively, TBI studies to date have reaffirmed that microglia are exquisitely sensitive to their microenvironment and complex with many fluctuating subpopulations, and that their reactivity can change based on the species, degree of injury, and over time.

## Sex differences in TBI

The overall TBI rate is greater in males compared to females. Among patients seen in Emergency Departments, 547.6 per 100,000 persons are males who experienced any-severity TBI, while 385.9 per 100,000 persons are female [68]. Interestingly, studies on collegiate athletes indicate that women experience more concussions than men when playing the same sport [69]. Moreover, a growing number of women are choosing to serve in active military duty, increasing their risk of combat-related injury [70].

To assess whether TBI outcomes are different in males vs females, Gupte et al. [71] performed an extensive review on sex differences in TBI in both human and animal research. Overall, the authors found that human studies have generally reported worse outcomes in women than men, whereas animal studies have generally reported better outcomes in females than males; however, the authors were careful to note that injury severity, genetic factors, and age may all interact with biological sex to determine TBI outcome [71]. Unfortunately, fewer women than men are enrolled in clinical trials for TBI, and male rodents have been predominately used in preclinical research [71]. The paucity of female subjects in brain injury research may impede the development of TBI treatments as biological sex can affect neuroanatomy, cellular pathways, and drug pharmacokinetics [72, 73]. In addition, sex hormones are an important aspect of effective TBI therapy as estrogen and progesterone have been examined for potential neuroprotective effects. Unfortunately, while preclinical studies have indicated a decrease in inflammation from progesterone treatment post-TBI, clinical trials have failed to demonstrate improved outcomes between progesterone treatment and placebo [74, 75].

Sex differences also exist in microglia quantity and phenotype according to recent rodent studies [75]. In early postnatal stages, males have more microglia in specific neuroanatomical subregions (preoptic area, parietal cortex, hippocampus, and amygdala) and generally have more ameboid microglia in the amygdala, while females have more ameboid microglia in the hippocampus (for a review see [76]). One study found that microglia from female brains at age 3 days expressed higher levels of inflammatory cytokines compared to male brains, yet differences did not exist at later timepoints [77]. Therefore, it has been postulated that microglia may have different roles and responses between males and females after TBI [75]. While the data are limited, several studies have examined differing microglia responses based on sex. After LPS stimulation, microglia in male mouse neonates expressed greater IL-1β compared to female neonates [78]. After penetrating brain injury, COX-2 expression (an enzyme which stimulates inflammation) and apoptotic cell death measured by TUNEL staining increased in male rats at 24 h post-injury, yet astrogliosis and microgliosis did not differ [79]. After a cortical stab wound, male mice had greater microglial density only at the lesion border [80]. Finally, after controlled cortical impact in adult mice, males showed a greater influx of peripheral myeloid cells followed by proliferation of microglia compared to females [81]. The data for sex differences in human microglia after TBI are lacking but the limited preclinical data underscores the importance of understanding TBI-related sex differences to develop effective clinical therapies.

Unfortunately, there is little data concerning sex differences after TBI in pigs [82]. One notable study conducted by Missios et al. [83] examined the effect of age and sex on lesion volume after cortical impact in piglets. Lesion volumes were significantly larger in male 1-month-old piglets and infant males had higher levels of circulating sex steroids compared to females. Future porcine TBI studies must enroll male and female subjects to understand sex differences in neuropathology and neuroinflammation, and to better serve female patients who have experienced TBI.

## Advantages and limitations of large animal models of mild TBI

Mild TBI accounts for 75% of all TBI incidents in the US each year and these injuries may cause long-term impairments or disabilities [84]. Mild TBI is generally distinguished from moderate and severe through diagnostic criteria; mild TBI can have any period of transient confusion and memory dysfunction around the time of injury, loss of consciousness lasting less than 30 min, a Glasgow coma scale score of 13–15 after 30 min, and computed tomography or magnetic resonance imaging may be normal [85, 86]. Therefore, preclinical TBI studies need to consider these diagnostic criteria as well as the biomechanics of human TBI to establish a viable translational model.

Prime candidates for recreating biomechanical parameters known to be injurious in humans are species with a large brain mass and gyrencephalic brain architecture. In particular, pigs are similar to humans in that they possess a relatively large brain mass and gyrencephalic convolutions with a 60:40 white:gray matter ratio—similar to the ratio found in the human brain [87]. This is in stark contrast to mice and rats typically used in TBI studies; mice and rats have smaller brain masses, smooth lissencephalic brains, and 10:90 and 14:86 white:gray matter ratios, respectively [87–89]. This is particularly important as white matter injury has been described as the hallmark pathology of closed-head diffuse TBI in humans [90].

To replicate the biomechanics of human TBI, our group conducts closed-head rotational injuries, the most common form of mild TBI and typically induced through rapid angular acceleration and deceleration of the head [1, 91, 92]. Pigs experienced peak angular acceleration (corresponding to maximum angular deceleration) ranging from 66,000 to 186,000 rad/s^2^ with an average minimum angular acceleration of 128,000 rad/s^2^. By scaling these angular accelerations according to the brain masses of pigs vs humans via Holburn’s scaling equation, we estimate that these injuries would be approximately equivalent to 10,000 rad/s^2^ to 29,000 rad/s^2^ (mean of approximately 23,000 rad/s^2^) in the human brain [93, 94]. These acceleration levels are similar to levels experienced by humans during TBI; on-field head impact data from high school and collegiate football players measure head angular acceleration ≤ 15,000 rad/s^2,^ while motor vehicle crash simulations predict head angular acceleration at approximately 25,000 rad/s^2^ [95–97].

Finally, many rodent studies use a contusion model that produces cortical tissue loss and cavitation that is not observed even in severe human TBI. Conversely, our pig model employs closed-head rotational acceleration TBI, which produces loss of consciousness from mild to severe levels and emulates the clinical criteria outlined for human mild TBI. The lack of rotational injury is a limitation in other large animal models of TBI as head rotation is critical in inducing loss of consciousness [98, 99]. Furthermore, our group has used these injury kinematics to examine acute recovery outcomes; compared to controls, pigs experiencing mild TBI were more likely to have apnea, shorter time to extubation, and longer time from extubation to standing [94]. Some rodent studies have tried to incorporate head acceleration, but rodent brains are simply too small to produce the appropriate rotation acceleration-induced forces and, therefore, cannot reach the scaled thresholds of injury [93, 100]. The scaled head rotational acceleration of our model, paired with the gyrencephalic architecture and proper white:gray matter ratio, allows us to produce pathology with the same anatomical patterns and distribution of human TBI, and, therefore, validates our large animal model of TBI according to biomechanical, clinical, and neuropathological criteria [56, 57, 101].

**Fig. 3:**
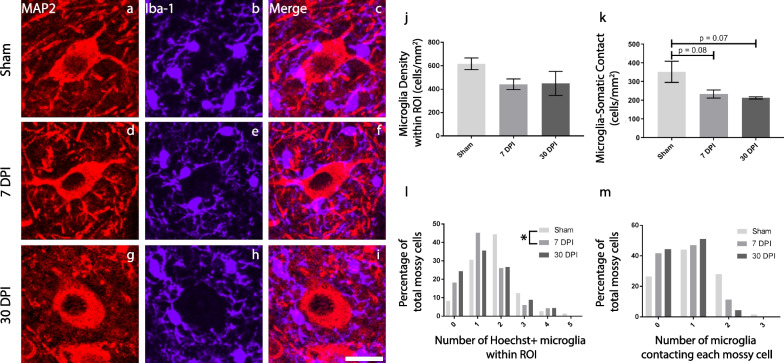
Microglia density proximal to hilar mossy cells changes following mild TBI. Representative images of hilar mossy cells from sham (**a**), 7-days post-injury (DPI) (**d**), and 30 DPI (**g**), microglial activity from sham (**b**), 7 DPI (**e**), and 30 DPI (**h**), and corresponding merged images (**c**, **f**, **i**) are shown (scale = 25 µm). Microglia density did not change significantly around mossy cells (**j**) but a histogram of the number of microglia per mossy cell indicates there are less microglia more frequently at 7 DPI compared to sham (**l**). There was a non-significant decrease in the number of microglia contacting mossy cell somata (**k**) and a non-significant leftward shift of the data distribution at 7 DPI and 30 DPI compared to sham (**m**) (Images adapted from Grovola et al. [104])

Using this large animal model of TBI, researchers have induced reproducible neurological and neuropathological deficits including loss of consciousness, mild edema, increased intracranial pressure, astrogliosis, diffuse axonal injury, and neuroinflammation [56, 57, 102, 103]. Our group has recently published a series of studies that examine neuropathology out to 1 year following a single mild TBI in male pigs. First, we analyzed synaptic changes and microglia activity in the hippocampus, where we found cell hypertrophy in hilar mossy cells—the primary glutamatergic neuron in the hilus—at 30 DPI, upregulation of synapsin labeling around mossy cells at 7 DPI, and an increase in microglia density at various subacute and chronic timepoints (Fig. 3) [104]. Then, to further characterize microglial phenotypes, we employed sensitive and quantitative morphological analysis of microglia using automated skeletal analysis. We observed increases to microglial ramification out to 1-year post-injury in various hippocampal sub-regions (Fig. 2h–k). We also noted an increase in microglial ramification in periventricular white matter at 3 DPI and 7 DPI, coinciding with an increase in axonal pathology in the periventricular white matter at these timepoints [67]. These data suggest that a single, closed-head mild TBI can produce persistent changes to the neuroimmune response.

**Fig. 4:**
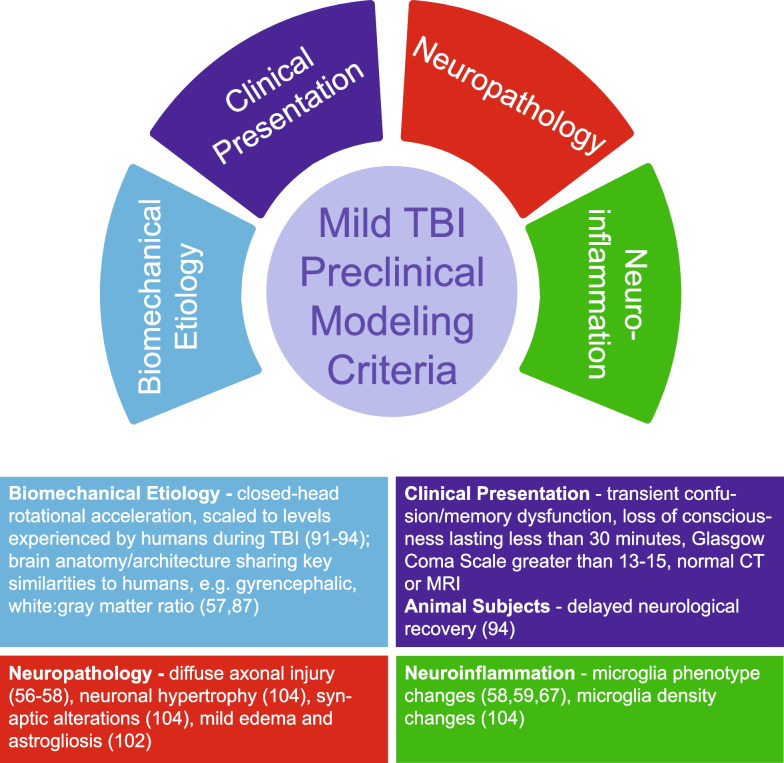
Mild TBI preclinical modeling criteria. After evaluation of numerous preclinical studies, as well as the clinical observations of mild TBI, we propose that preclinical models of mild TBI be validated through biomechanical, clinical, neuropathological, and neuroinflammatory criteria

However, further experimentation, such as gene expression changes and antibody labeling for acute and chronic neuroinflammatory cytokines, is needed to provide more contextual detail to microglial morphological changes and provide knowledge of their role in neuronal health and synaptic function post TBI. Absent these additional studies, interpretation of these phenotypic microglia changes is limited. Morphological changes to microglia suggest a change in neuroimmune homeostasis, which may be driven by numerous extracellular signals, such as disrupted BBB or damaged neurons. While we have not observed overt neuronal loss in this model, microglia may be monitoring injured neurons and stabilizing their synaptic function [104]. Based on these observed chronic microglial phenotype changes in our validated model of injury, we propose that preclinical models of mild TBI also be validated on neuroinflammatory criteria, in addition to clinical, biomechanical, and neuropathological (Fig. 4).

Large animal models do have distinct disadvantages, however. Large animal subjects themselves cost much more than rodents, as does the necessary specialized housing. Veterinary staff needs to be trained for larger animal handling and care, which may impact feasibility. In addition, post-TBI behavioral and cognitive tests are not thoroughly validated in a porcine model and many commercially available antibodies do not cross-react with porcine tissue, thus restricting some experimental measures. Finally, genetic and mechanistic modulations are much more difficult in porcine models compared to rodent models. As such, rodent and large animal models may have complementary roles as the field seeks to examine neuroinflammation after TBI and better understand the chronic consequences of brain injury.

## Prospects for therapy

Post-traumatic neuroinflammation provides vital physiological functions as we have outlined in previous sections. However, this response can be pushed beyond homeostatic parameters or may continue beyond the acute injury period, potentially contributing to a lifetime of disability and neurodegenerative disease. Unfortunately, targeting and treating inflammation after TBI has proven challenging as many clinical trial have failed that directly or indirectly influenced inflammatory processes, including corticosteroids, hypothermia, and hypertonic saline infusion [105–108]. One anti-inflammatory clinical trial had mixed results; minocycline administration after TBI in 15 patients reduced chronic microglial activation but increased neurodegeneration [109].

As clinical trials continue to optimize the neuroimmune response following TBI, a new intervention framework has been proposed by Simon et al. [8]: limit the acute proinflammatory response to essential debris clearance and danger signals, promote anti-inflammatory and pro-regenerative immune cells, and prevent chronic inflammation. This framework suggests that specific components of neuroinflammation should be modulated in a time dependent manner. For instance, due to the role of the complement system in enhancing phagocytic activity and inflammation after TBI, blocking this cascade has become an attractive therapeutic strategy. Blocking different steps in the complement cascade has mitigated neuronal loss and chronic inflammation in rodent models of TBI [110, 111].

Another method that allows finer control over microglia involves inhibiting the microglia colony stimulating factor 1 receptor (CSF1R), a receptor expressed by microglia, macrophages, and osteoclasts, which can deplete the brain’s microglia population when the gene is eliminated [112, 113]. One study of note administered mice a CSF1R inhibitor for 1 week at 28-days post-moderate TBI, which improved motor and cognitive function, decreased lesion volume, and attenuated cortical and dentate gyrus lesion loss [62]. Even though these data are promising, many other factors will need to be assessed to determine the true effectiveness of therapeutics for post-TBI inflammation. Primarily, all immune cell types should be carefully assessed as one study suggests that CSF1R inhibitors also affect peripheral immune cells and inhibit circulating tissue macrophages [114]. In addition, candidate therapeutics should carefully consider various factors that may affect the neuroimmune response to TBI, such as age, sex, mechanism and degree of injury, and secondary insults, with the overall goal of promoting regeneration and mitigating degeneration following TBI.

## Conclusions

Microglia are necessary immune cells in the CNS that may contribute to both neuroprotection and neurodegeneration after TBI. Microglia can be driven by a variety of extracellular signals and microglial activation is often sustained out to chronic timepoints. Preclinical modeling of TBI-induced neuroinflammation is a challenge as injury severity, species, and biological sex are among the many factors than can influence the inflammatory response. However, the most translatable preclinical research will strive to mirror human TBI according to neuroinflammatory responses, in addition to the three classic benchmarks of biomechanical etiology, clinical presentation, and neuropathological sequelae. Further research is needed to elucidate the potential benefits and/or neurotoxic effects of microglia after TBI. Only a multipronged approach of genomic analysis, pharmacological intervention, neuroimaging, and behavioral assays will improve our understanding of the chronic neuroinflammatory sequela to TBI and advance treatment options for patients that suffer from deleterious post-TBI effects.

## Data Availability

Not applicable.

## Abbreviations

TBI: Traumatic brain injury
CNS: Central nervous system
LTP: Long-term potentiation
TNF: Tumor necrosis factor
DAMPs: Damaged-associated molecular patterns
HMGB1: High mobility group protein B1
ATP: Adenosine 5′-triphosphate
TLR: Toll-like receptors
NLR: NOD-like receptors
NMDA: *N*-Methyl-d-aspartate
AMPA: α-Amino-3-hydroxy-5-methyl-4-isoxazolepropionic acid
PET: Positron emission tomography
DPI: Days post-injury
CSF1R: Colony stimulating factor 1 receptor

## References

[1] Centers for Disease Control and Prevention. Surveillance report of traumatic brain injury-related emergency department visits, hospitalizations, and deaths-United States, 2014. Centers for Disease Control and Prevention, US Department of Health and Human Services. 2019;24. www.cdc.gov/TraumaticBrainInjury.

[2] Loane DJ, Faden AI (2010). Neuroprotection for traumatic brain injury: translational challenges and emerging therapeutic strategies. Trends Pharmacol Sci.

[3] Ekmark-Lewén S, Flygt J, Kiwanuka O, Meyerson BJ, Lewén A, Hillered L (2013). Traumatic axonal injury in the mouse is accompanied by a dynamic inflammatory response, astroglial reactivity and complex behavioral changes. J Neuroinflamm.

[4] Singleton RH, Povlishock JT (2004). Identification and characterization of heterogeneous neuronal injury and death in regions of diffuse brain injury: evidence for multiple independent injury phenotypes. J Neurosci.

[5] Johnson VE, Stewart JE, Begbie FD, Trojanowski JQ, Smith DH, Stewart W (2013). Inflammation and white matter degeneration persist for years after a single traumatic brain injury. Brain.

[6] Karve IP, Taylor JM, Crack PJ (2015). The contribution of astrocytes and microglia to traumatic brain injury. Br J Pharmacol.

[7] Jassam YN, Izzy S, Whalen M, McGavern DB, El Khoury J (2017). Neuroimmunology of traumatic brain injury: time for a paradigm shift. Neuron.

[8] Simon DW, McGeachy MJ, Bayır H, Clark RSBB, Loane DJ, Kochanek PM (2017). The far-reaching scope of neuroinflammation after traumatic brain injury. Nat Rev Neurol.

[9] Ginhoux F, Greter M, Leboeuf M, Nandi S, See P, Gokhan S (2010). Fate mapping analysis reveals that adult microglia derive from primitive macrophages. Science (80-).

[10] Schulz C, Perdiguero EG, Chorro L, Szabo-Rogers H, Cagnard N, Kierdorf K (2012). A lineage of myeloid cells independent of myb and hematopoietic stem cells. Science (80-).

[11] Davalos D, Grutzendler J, Yang G, Kim JV, Zuo Y, Jung S (2005). ATP mediates rapid microglial response to local brain injury in vivo. Nat Neurosci.

[12] Nimmerjahn A, Kirchhoff F, Helmchen F (2005). Resting microglial cells are highly dynamic surveillants of brain parenchyma in vivo. Neuroforum.

[13] Schafer DP, Lehrman EK, Kautzman AG, Koyama R, Mardinly AR, Yamasaki R (2012). Microglia sculpt postnatal neural circuits in an activity and complement-dependent manner. Neuron.

[14] Paolicelli RC, Bolasco G, Pagani F, Maggi L, Scianni M, Panzanelli P (2011). Synaptic pruning by microglia is necessary for normal brain development. Science (80-).

[15] Weinhard L, di Bartolomei G, Bolasco G, Machado P, Schieber NL, Neniskyte U (2018). Microglia remodel synapses by presynaptic trogocytosis and spine head filopodia induction. Nat Commun.

[16] Sipe GO, Lowery RL, Tremblay M, Kelly EA, Lamantia CE, Majewska AK (2016). Microglial P2Y12 is necessary for synaptic plasticity in mouse visual cortex. Nat Commun.

[17] Nguyen PT, Dorman LC, Pan S, Vainchtein ID, Han RT, Nakao-Inoue H (2020). Microglial remodeling of the extracellular matrix promotes synapse plasticity. Cell.

[18] Rogers JT, Morganti JM, Bachstetter AD, Hudson CE, Peters MM, Grimmig BA (2011). CX3CR1 deficiency leads to impairment of hippocampal cognitive function and synaptic plasticity. J Neurosci.

[19] Sierra A, Encinas JM, Deudero JJP, Chancey JH, Enikolopov G, Overstreet-Wadiche LS (2010). Microglia shape adult hippocampal neurogenesis through apoptosis-coupled phagocytosis. Cell Stem Cell.

[20] Brown GC, Neher JJ (2014). Microglial phagocytosis of live neurons. Nat Rev Neurosci.

[21] Fourgeaud L, Través PG, Tufail Y, Leal-Bailey H, Lew ED, Burrola PG (2016). TAM receptors regulate multiple features of microglial physiology. Nature.

[22] Sedel F, Béchade C, Vyas S, Triller A (2004). Macrophage-derived tumor necrosis factor alpha, an early developmental signal for motoneuron death. J Neurosci.

[23] Marín-Teva JL, Dusart I, Colin C, Gervais A, van Rooijen N, Mallat M (2004). Microglia promote the death of developing Purkinje cells. Neuron.

[24] Salter MW, Stevens B (2017). Microglia emerge as central players in brain disease. Nat Med.

[25] Ransohoff RM (2016). A polarizing question: do M1 and M2 microglia exist. Nat Neurosci.

[26] Morganti JM, Riparip L-KK, Rosi S (2016). Call off the Dog(ma): M1/M2 polarization is concurrent following traumatic brain injury. PLoS ONE.

[27] Paolicelli R, Sierra A, Stevens B, Tremblay M-E, Aguzzi A, Ajami B, et al. Defining microglial states and nomenclature: a roadmap to 2030. SSRN Electron J. 2022. https://www.ssrn.com/abstract=4065080.

[28] Hammond TR, Dufort C, Dissing-Olesen L, Giera S, Young A, Wysoker A (2019). Single-cell RNA sequencing of microglia throughout the mouse lifespan and in the injured brain reveals complex cell-state changes. Immunity.

[29] Shih BB, Brown SM, Barrington J, Lefevre L, Mabbott NA, Priller J (2022). Defining the pig microglial transcriptome reveals its core signature, regional heterogeneity, and similarity with human and rodent microglia. Glia.

[30] Wofford KL, Loane DJ, Cullen DK (2019). Acute drivers of neuroinflammation in traumatic brain injury. Neural Regen Res.

[31] Laird MD, Shields JS, Sukumari-Ramesh S, Kimbler DE, Fessler RD, Shakir B (2014). High mobility group box protein-1 promotes cerebral edema after traumatic brain injury via activation of toll-like receptor 4. Glia.

[32] Eyo UB, Wu L-J (2013). Bidirectional microglia-neuron communication in the healthy brain. Neural Plast.

[33] Bianco F, Pravettoni E, Colombo A, Schenk U, Möller T, Matteoli M (2005). Astrocyte-derived ATP induces vesicle shedding and IL-1β release from microglia. J Immunol.

[34] Ransohoff RM, Brown MA (2012). Innate immunity in the central nervous system. J Clin Invest.

[35] Kerr N, de Rivero Vaccari JP, Dietrich WD, Keane RW (2020). Neural-respiratory inflammasome axis in traumatic brain injury. Exp Neurol.

[36] O’Brien WT, Pham L, Symons GF, Monif M, Shultz SR, McDonald SJ (2020). The NLRP3 inflammasome in traumatic brain injury: potential as a biomarker and therapeutic target. J Neuroinflamm.

[37] Yi JH, Hazell AS (2006). Excitotoxic mechanisms and the role of astrocytic glutamate transporters in traumatic brain injury. Neurochem Int.

[38] Cantu D, Walker K, Andresen L, Taylor-Weiner A, Hampton D, Tesco G (2015). Traumatic brain injury increases cortical glutamate network activity by compromising GABAergic control. Cereb Cortex.

[39] Kochanek PM, Clark RSB, Ruppel RA, Adelson PD, Bell MJ, Whalen MJ (2000). Biochemical, cellular, and molecular mechanisms in the evolution of secondary damage after severe traumatic brain injury in infants and children: lessons learned from the bedside. Pediatr Crit Care Med.

[40] Noda M, Nakanishi H, Nabekura J, Akaike N (2000). AMPA-kainate subtypes of glutamate receptor in rat cerebral microglia. J Neurosci.

[41] Taylor DL, Diemel LT, Cuzner ML, Pocock JM (2002). Activation of group II metabotropic glutamate receptors underlies microglial reactivity and neurotoxicity following stimulation with chromogranin A, a peptide up-regulated in Alzheimer’s disease. J Neurochem.

[42] Taylor DL, Diemel LT, Pocock JM (2003). Activation of microglial group III metabotropic glutamate receptors protects neurons against microglial neurotoxicity. J Neurosci.

[43] Taylor DL, Jones F, Kubota ESFCS, Pocock JM (2005). Stimulation of microglial metabotropic glutamate receptor mGlu2 triggers tumor necrosis factor alpha-induced neurotoxicity in concert with microglial-derived Fas ligand. J Neurosci.

[44] Viviani B, Boraso M, Marchetti N, Marinovich M (2014). Perspectives on neuroinflammation and excitotoxicity: a neurotoxic conspiracy?. Neurotoxicology.

[45] Ikonomidou C, Turski L (2002). Why did NMDA receptor antagonists fail clinical trials for stroke and traumatic brain injury?. Lancet Neurol.

[46] Smith C, Gentleman SM, Leclercq PD, Murray LS, Griffin WST, Graham DI (2013). The neuroinflammatory response in humans after traumatic brain injury. Neuropathol Appl Neurobiol.

[47] Loane DJ, Kumar A, Stoica BA, Cabatbat R, Faden AI (2014). Progressive neurodegeneration after experimental brain trauma. J Neuropathol Exp Neurol.

[48] Coughlin JM, Wang Y, Minn I, Bienko N, Ambinder EB, Xu X (2017). Imaging of glial cell activation and white matter integrity in brains of active and recently retired national football league players. JAMA Neurol.

[49] Juengst SB, Kumar RG, Arenth PM, Wagner AK (2014). Exploratory associations with tumor necrosis factor-α, disinhibition and suicidal endorsement after traumatic brain injury. Brain Behav Immun.

[50] Witcher KG, Bray CE, Chunchai T, Zhao F, O’Neil SM, Gordillo AJ (2021). Traumatic brain injury causes chronic cortical inflammation and neuronal dysfunction mediated by microglia. J Neurosci.

[51] Todd BP, Chimenti MS, Luo Z, Ferguson PJ, Bassuk AG, Newell EA (2021). Traumatic brain injury results in unique microglial and astrocyte transcriptomes enriched for type I interferon response. J Neuroinflamm.

[52] Lipponen A, Paananen J, Puhakka N, Pitkanen A (2016). Analysis of post-traumatic brain injury gene expression signature reveals tubulins, Nfe2l2, Nfkb, Cd44, and S100a4 as treatment targets. Sci Rep.

[53] Friess SH, Ralston J, Eucker SA, Helfaer MA, Smith C, Margulies SS (2011). Neurocritical care monitoring correlates with neuropathology in a swine model of pediatric traumatic brain injury. Neurosurgery.

[54] Margulies SS, Kilbaugh T, Sullivan S, Smith C, Propert K, Byro M (2015). Establishing a clinically relevant large animal model platform for TBI therapy development: using cyclosporin a as a case study. Brain Pathol.

[55] Mayer AR, Ling JM, Dodd AB, Rannou-Latella JG, Stephenson DD, Dodd RJ (2021). Reproducibility and characterization of head kinematics during a large animal acceleration model of traumatic brain injury. Front Neurol.

[56] Smith DH, Nonaka M, Miller R, Leoni M, Chen X-H, Alsop D (2000). Immediate coma following inertial brain injury dependent on axonal damage in the brainstem. J Neurosurg.

[57] Cullen DK, Harris JP, Browne KD, Wolf JA, Duda JE, Meaney DF (2016). A porcine model of traumatic brain injury via head rotational acceleration. Methods Mol Biol.

[58] Lafrenaye AD, Todani M, Walker SA, Povlishock JT (2015). Microglia processes associate with diffusely injured axons following mild traumatic brain injury in the micro pig. J Neuroinflamm.

[59] Wofford KL, Harris JP, Browne KD, Brown DP, Grovola MR, Mietus CJ (2017). Rapid neuroinflammatory response localized to injured neurons after diffuse traumatic brain injury in swine. Exp Neurol.

[60] Kumar A, Stoica BA, Sabirzhanov B, Burns MP, Faden AI, Loane DJ (2013). Traumatic brain injury in aged animals increases lesion size and chronically alters microglial/macrophage classical and alternative activation states. Neurobiol Aging.

[61] Hiskens MI, Schneiders AG, Vella RK, Fenning AS (2021). Repetitive mild traumatic brain injury affects inflammation and excitotoxic mRNA expression at acute and chronic time-points. PLoS ONE.

[62] Henry RJ, Ritzel RM, Barrett JP, Doran SJ, Jiao Y, Leach JB (2020). Microglial depletion with CSF1R inhibitor during chronic phase of experimental traumatic brain injury reduces neurodegeneration and neurological deficits. J Neurosci.

[63] Gorse KM, Lafrenaye AD (2018). The importance of inter-species variation in traumatic brain injury-induced alterations of microglial-axonal interactions. Front Neurol.

[64] Hay J, Johnson VE, Smith DH, Stewart W (2016). Chronic traumatic encephalopathy: the neuropathological legacy of traumatic brain injury. Annu Rev Pathol Mech Dis.

[65] McKee AC, Stein TD, Kiernan PT, Alvarez VE (2015). The neuropathology of chronic traumatic encephalopathy. Brain Pathol.

[66] Ramlackhansingh AF, Brooks DJ, Greenwood RJ, Bose SK, Turkheimer FE, Kinnunen KM (2011). Inflammation after trauma: microglial activation and traumatic brain injury. Ann Neurol.

[67] Grovola MR, Paleologos N, Brown DP, Tran N, Wofford KL, Harris JP (2021). Diverse changes in microglia morphology and axonal pathology during the course of 1 year after mild traumatic brain injury in pigs. Brain Pathol.

[68] Faul M, Coronado V (2015). Epidemiology of traumatic brain injury. Handbook of clinical neurology.

[69] Covassin T, Moran R, Elbin RJ (2016). Sex differences in reported concussion injury rates and time loss from participation: an update of the national collegiate athletic association injury surveillance program from 2004–2005 through 2008–2009. J Athl Train.

[70] Amoroso T, Iverson KM (2017). Acknowledging the risk for traumatic brain injury in women veterans. J Nerv Ment Dis.

[71] Gupte R, Brooks W, Vukas R, Pierce J, Harris J (2019). Sex differences in traumatic brain injury: what we know and what we should know. J Neurotrauma.

[72] McCarthy MM, Pickett LA, VanRyzin JW, Kight KE (2015). Surprising origins of sex differences in the brain. Horm Behav.

[73] Soldin OP, Mattison DR (2009). Sex differences in pharmacokinetics and pharmacodynamics. Clin Pharmacokinet.

[74] Lin C, He H, Li Z, Liu Y, Chao H, Ji J (2015). Efficacy of progesterone for moderate to severe traumatic brain injury: a meta-analysis of randomized clinical trials. Sci Rep.

[75] Caplan HW, Cox CS, Bedi SS (2017). Do microglia play a role in sex differences in TBI?. J Neurosci Res.

[76] Bordt EA, Ceasrine AM, Bilbo SD (2020). Microglia and sexual differentiation of the developing brain: a focus on ontogeny and intrinsic factors. Glia.

[77] Crain JM, Nikodemova M, Watters JJ (2013). Microglia express distinct M1 and M2 phenotypic markers in the postnatal and adult central nervous system in male and female mice. J Neurosci Res.

[78] Loram LC, Sholar PW, Taylor FR, Wiesler JL, Babb JA, Strand KA (2012). Sex and estradiol influence glial pro-inflammatory responses to lipopolysaccharide in rats. Psychoneuroendocrinology.

[79] Günther M, Plantman S, Davidsson J, Angéria M, Mathiesen T, Risling M (2015). COX-2 regulation and TUNEL-positive cell death differ between genders in the secondary inflammatory response following experimental penetrating focal brain injury in rats. Acta Neurochir (Wien).

[80] Acaz-Fonseca E, Duran JC, Carrero P, Garcia-Segura LM, Arevalo MA (2015). Sex differences in glia reactivity after cortical brain injury. Glia.

[81] Doran SJ, Ritzel RM, Glaser EP, Henry RJ, Faden AI, Loane DJ (2019). Sex differences in acute neuroinflammation after experimental traumatic brain injury are mediated by infiltrating myeloid cells. J Neurotrauma.

[82] Rubin TG, Lipton ML (2019). Sex differences in animal models of traumatic brain injury. J Exp Neurosci.

[83] Missios S, Harris BT, Dodge CP, Simoni MK, Costine BA, Lee YL (2009). Scaled cortical impact in immature swine: effect of age and gender on lesion volume. J Neurotrauma.

[84] National Center for Injury Prevention and Control. Report to congress on mild traumatic brain injury in the United States: steps to prevent a serious public health problem. Atlanta, GA; 2003. http://www.cdc.gov/traumaticbraininjury/pdf/mtbireport-a.pdf.

[85] Menon DK, Schwab K, Wright DW, Maas AI (2010). Position statement: definition of traumatic brain injury. Arch Phys Med Rehabil.

[86] Kay T, Harrington DE, Adams R, Anderson T, Berrol S, Cicerone K (1993). Definition of mild traumatic brain injury. J Head Trauma Rehabil.

[87] Zhang K, Sejnowski TJ (2000). A universal scaling law between gray matter and white matter of cerebral cortex. Proc Natl Acad Sci.

[88] Bailey EL, Mcculloch J, Sudlow C, Wardlaw JM (2009). Potential animal models of lacunar stroke: a systematic review. Stroke.

[89] Howells DW, Porritt MJ, Rewell SSJ, O’Collins V, Sena ES, Van Der Worp HB (2010). Different strokes for different folks: the rich diversity of animal models of focal cerebral ischemia. J Cereb Blood Flow Metab.

[90] Johnson VE, Stewart W, Smith DH (2013). Axonal pathology in traumatic brain injury. Exp Neurol.

[91] Meaney DF, Olvey SE, Gennarelli TA (2011). Biomechanical basis of traumatic brain injury. Youmans neurological surgery.

[92] LaPlaca MC, Simon CM, Prado GR, Cullen DK (2007). CNS injury biomechanics and experimental models. Prog Brain Res.

[93] Holbourn AHS (1943). Mechanics of head injuries. Lancet.

[94] Wofford KL, Grovola MR, Adewole DO, Browne KD, Putt ME, O’Donnell JC (2021). Relationships between injury kinematics, neurological recovery, and pathology following concussion. Brain Commun.

[95] Greenwald RM, Gwin JT, Chu JJ, Crisco JJ (2008). Head impact severity measures for evaluating mild traumatic brain injury risk exposure. Neurosurgery.

[96] Crisco JJ, Wilcox BJ, Beckwith JG, Chu JJ, Duhaime A-C, Rowson S (2011). Head impact exposure in collegiate football players. J Biomech.

[97] Ahmadisoleymani SS, Missoum S (2019). Construction of a risk model through the fusion of experimental data and finite element modeling: application to car crash-induced TBI. Comput Methods Biomech Biomed Eng.

[98] Vink R (2018). Large animal models of traumatic brain injury. J Neurosci Res.

[99] Gennarelli TA, Thibault LE, Adams JH, Graham DI, Thompson CJ, Marcincin RP (1982). Diffuse axonal injury and traumatic coma in the primate. Ann Neurol.

[100] Sauerbeck AD, Fanizzi C, Kim JH, Gangolli M, Bayly PV, Wellington CL (2018). modCHIMERA: a novel murine closed-head model of moderate traumatic brain injury. Sci Rep.

[101] Browne KD, Chen X-H, Meaney DF, Smith DH (2011). Mild traumatic brain injury and diffuse axonal injury in swine. J Neurotrauma.

[102] Smith DH, Chen XH, Xu BN, Mcintosh TK, Gennarelli TA, Meaney DF (1997). Characterization of diffuse axonal pathology and selective hippocampal damage following inertial brain trauma in the pig. J Neuropathol Exp Neurol.

[103] Chen X, Siman R, Iwata A, Meaney DF, Trojanowski JQ, Smith DH (2004). Long-term accumulation of amyloid-β, β-secretase, presenilin-1, and caspase-3 in damaged axons following brain trauma. Am J Pathol.

[104] Grovola MR, Paleologos N, Wofford KL, Harris JP, Browne KD, Johnson V (2020). Mossy cell hypertrophy and synaptic changes in the hilus following mild diffuse traumatic brain injury in pigs. J Neuroinflamm.

[105] Asehnoune K, Seguin P, Allary J, Feuillet F, Lasocki S, Cook F (2014). Hydrocortisone and fludrocortisone for prevention of hospital-acquired pneumonia in patients with severe traumatic brain injury (Corti-TC): a double-blind, multicentre phase 3, randomised placebo-controlled trial. Lancet Respir Med.

[106] Roberts I, Yates D, Sandercock P, Farrell B, Wasserberg J, Lomas G (2004). Effect of intravenous corticosteroids on death within 14 days in 10008 adults with clinically significant head injury (MRC CRASH trial): randomised placebo-controlled trial. Lancet.

[107] Hutchison JS, Ward RE, Lacroix J, Hébert PC, Barnes MA, Bohn DJ (2008). Hypothermia therapy after traumatic brain injury in children. N Engl J Med.

[108] Bulger EM, May S, Brasel KJ, Schreiber M, Kerby JD, Tisherman SA (2010). Out-of-hospital hypertonic resuscitation following severe traumatic brain injury. JAMA.

[109] Scott G, Zetterberg H, Jolly A, Cole JH, De SS, Jenkins PO (2017). Minocycline reduces chronic microglial activation after brain trauma but increases neurodegeneration. Brain.

[110] Alawieh A, Langley EF, Weber S, Adkins D, Tomlinson S (2018). Identifying the role of complement in triggering neuroinflammation after traumatic brain injury. J Neurosci.

[111] Fluiter K, Opperhuizen AL, Morgan BP, Baas F, Ramaglia V (2014). Inhibition of the membrane attack complex of the complement system reduces secondary neuroaxonal loss and promotes neurologic recovery after traumatic brain injury in mice. J Immunol.

[112] Patel S, Player MR (2009). Colony-stimulating factor-1 receptor inhibitors for the treatment of cancer and inflammatory disease. Curr Top Med Chem.

[113] Erblich B, Zhu L, Etgen AM, Dobrenis K, Pollard JW (2011). Absence of colony stimulation factor-1 receptor results in loss of microglia, disrupted brain development and olfactory deficits. PLoS ONE.

[114] Lei F, Cui N, Zhou C, Chodosh J, Vavvas DG, Paschalis EI (2020). CSF1R inhibition by a small-molecule inhibitor is not microglia specific; affecting hematopoiesis and the function of macrophages. Proc Natl Acad Sci USA.

